# Single-Color Isomer-Resolved Spectroscopy

**DOI:** 10.1021/acs.jpca.2c02277

**Published:** 2022-06-01

**Authors:** Grite L. Abma, Dries Kleuskens, Siwen Wang, Michiel Balster, Andre van Roij, Niek Janssen, Daniel A. Horke

**Affiliations:** † Radboud University, Institute for Molecules and Materials, Heijendaalseweg 135, 6525 AJ Nijmegen, The Netherlands

## Abstract

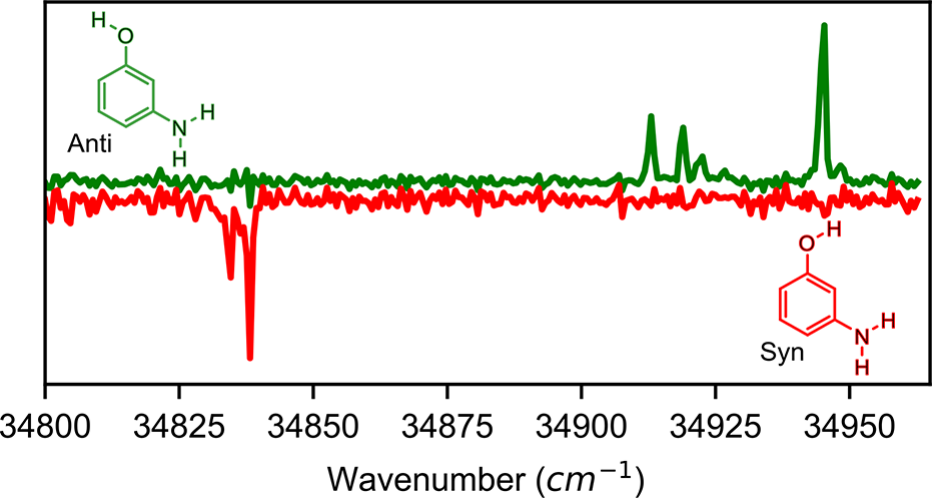

Structural isomers, such as conformers or tautomers, are of significant importance across chemistry and biology, as they can have different functionalities. In gas-phase experiments using molecular beams, formation of many different isomers cannot be prevented, and their presence significantly complicates the assignment of spectral lines. Current isomer-resolved spectroscopic techniques heavily rely on theoretical calculations or make use of elaborate double-resonance schemes. We show here that isomer-resolved spectroscopy can also be performed using a single tunable laser. In particular, we demonstrate single-color isomer-resolved spectroscopy by utilizing electrostatic deflection to spatially separate the isomers. We show that for 3-aminophenol we can spatially separate the *syn* and *anti* conformers and use these pure samples to perform high-resolution REMPI spectroscopy, making the assignment of transitions to a particular isomer trivial, without any additional *a priori* information. This approach allows one to add isomer specificity to any molecular-beam-based experiment.

## I Introduction

Chemical and biological functionality is defined through the underlying molecular structure. A special part of this structure–function relationship is the role of conformational isomerism, that is, systems that can interconvert through the rotation around a single bond. This seemingly trivial structural change can have far-reaching consequences and can lead to, for example, different chemical reactivities^1,2^ or different ordering of macromolecular structures such as proteins.^3^

The occurrence of different conformers has received considerable attention with the advent of high-resolution gas-phase spectroscopy based on supersonic molecular beams. Under the collision-free and rotationally cold conditions in these sources, different conformations do not interconvert and can be considered separate molecular species. Even for small organic systems, many conformers are frequently observed in the gas phase, for example, the smallest amino acid glycine has been produced in at least four different gas-phase conformations, with the exact number still subject to intense debate.^4−6^ This wide conformational sampling can be considered a blessing and a curse. The presence of many structures allows for detailed studies of intramolecular interactions and also aids in linking gas-phase spectroscopy to condensed-phase systems, where typically one conformation dominates due to intermolecular interactions with the solvent. However, extracting spectroscopic information for one particular conformation present in the supersonic expansion and assigning this to a specific molecular structure is not trivial and typically relies on *ab initio* theory.

The current experimental approach to conformer-resolved spectroscopy is based on double-resonance hole-burning techniques.^7^ These rely on having one resonant laser to selectively ionize the ground state of a specific conformer and a second laser to drive a transition that depletes (“burns”) the ground state. The burn laser is fired first, and scanning this thus allows recording a conformer-resolved spectrum, which in favorable cases can be assigned to a specific molecular geometry with the aid of theoretical chemistry calculations. This approach, and variations thereof, is now widely used across both the IR and UV spectral regions.^8−12^

**Figure 1:**
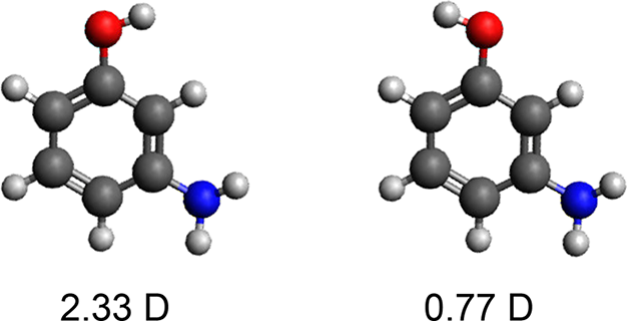
Structures and permanent dipole moments of *syn* and *anti* 3-aminophenol.

We present here an alternative approach to record conformer-resolved electronic spectra that does not rely on double-resonance techniques and does not require the calculation of theoretical spectra. In particular, we show that we can make use of the differences in the permanent dipole moment of conformers to fully separate them using electrostatic fields^13,14^ and then record single-color resonance enhanced multiphoton ionization (REMPI) spectra of the conformer-pure sample. This allows the trivial and unambiguous assignment of the observed spectral lines to a molecular structure. We demonstrate this here for the *syn* and *anti*-conformers of 3-aminophenol, which have a difference in dipole moment of around 1.5 D, as shown in Figure 1. Our methodology allowed us to observe and assign several torsional modes of this system for the first time. The presented approach is generally applicable to all molecular systems where the conformations exhibit a sufficient difference in dipole moment and can add conformer specificity to any molecular-beam-based spectroscopy experiment.

## II Methods

3-Aminophenol (98% purity, Sigma-Aldrich) was used without further purification and introduced into a molecular beam through seeding in 28 bar of neon and expansion in a pulsed Even Lavie valve,^15^ operated at 500 Hz and heated to 140 °C. The molecular beam was skimmed twice before entering a 300-mm-long electrostatic deflector. This had a rod-and-through-type electrode geometry in which the electrodes were separated by a distance of 3.4 mm.^16^ The electrodes were designed such that the field gradient is nearly constant in the vertical direction while being almost zero in the horizontal direction. Polar molecules flying through the deflector therefore experience a force in the vertical direction, depending on their effective dipole moment.^14^ The deflector was operated at potential differences up to 15 kV, corresponding to a maximum field gradient of around 50 kV/cm. Following the deflector, the molecular beam was skimmed once more before entering the interaction chamber. Further technical details are given in the Supporting Information. In the detection region, molecules were ionized via 1 + 1 resonance enhanced multiphoton ionization (REMPI), using the frequency doubled output of a Nd:YAG pumped dye laser (Pyrromethene-597 dye pumped at 532 nm), providing 7 ns pulses that were attenuated to 6 μJ per pulse, and operating at 50 Hz. The laser was focused using an *f* = 750 mm lens to a spot size of approximately 100 μm inside the center of a three-plate velocity-map imaging spectrometer,^17^ operated here in ion time-of-flight mode. Spatial distributions of the molecular beam were measured by translating the laser focus through the molecular beam.

Experimental deflection measurements were complemented by trajectory simulations. For these, the Stark effect of both conformers was calculated using the freely available CMIstark software package.^18^ Trajectories of individual quantum states (*J* = 0–15, 1000 trajectories per state) were then propagated through the experimental setup. Individual trajectories were combined to a simulated deflection profile through Boltzmann weighting, with the rotational temperature fitted to the experimental data.^14^

## III Results and Discussion

**Figure 2:**
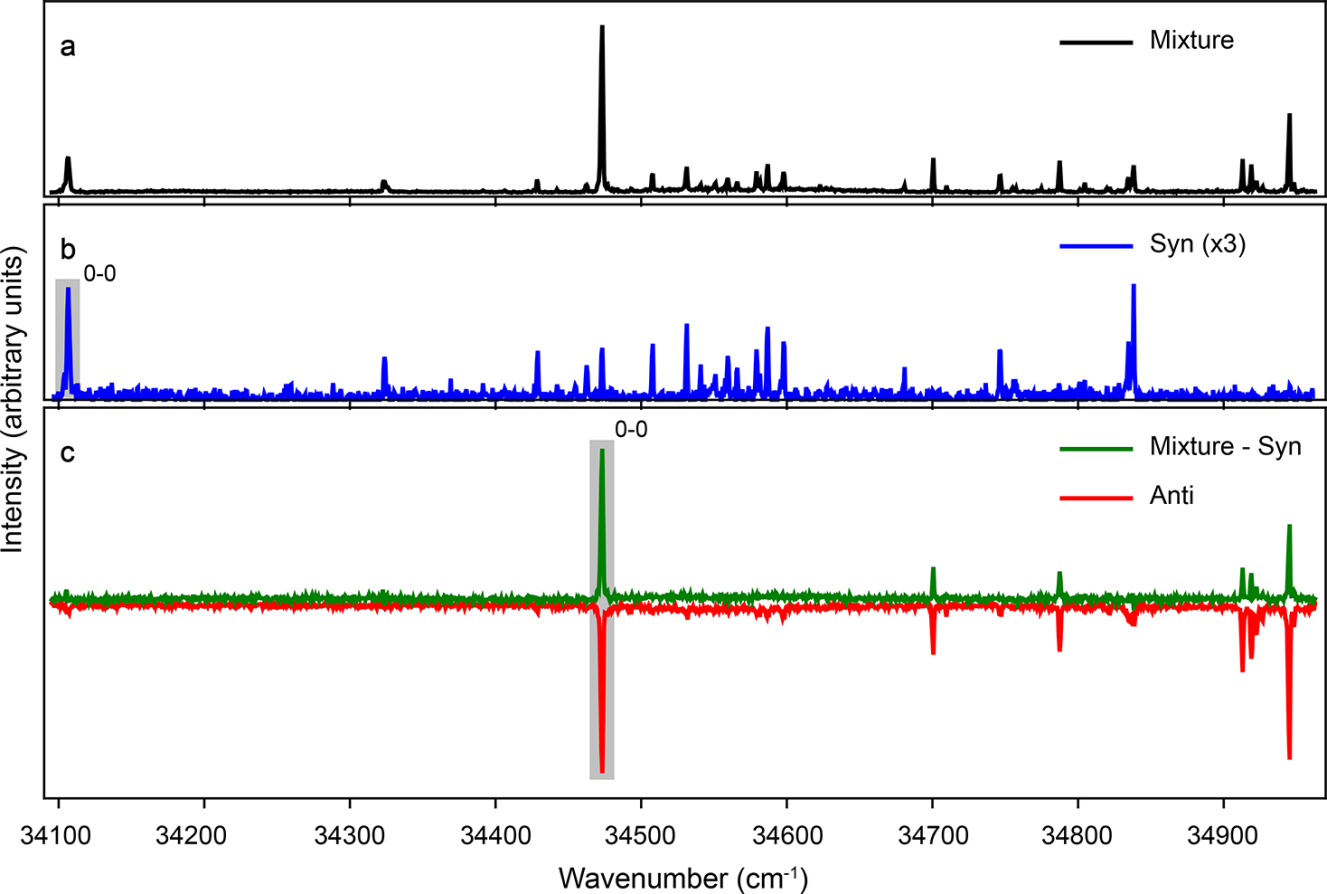
Resonance enhanced multiphoton ionization spectra of 3-aminophenol. (a) Measured in the middle of the molecular beam without deflection fields, containing a mixture of *syn* and *anti* conformers. (b) At the most deflected edge of the molecular beam, containing a pure sample of *syn* 3-aminophenol. (c) Mixture spectrum with the pure *syn* spectrum subtracted (green), as well as the spectrum collected at the undeflected edge containing a nearly pure sample of *anti* conformers (red).

A conventional single-color 1 + 1 REMPI spectrum of 3-aminophenol from 34 100 to 35 000 cm^–1^ is shown at the top of Figure 2. This is consistent with previously reported REMPI spectra,^19^ with band origins for the *syn* and *anti* conformers at 34 106.8 and 34 473.3 cm^–1^, respectively. Assuming identical efficiencies of the REMPI process for both conformers, the origin peaks yield a syn/anti ratio of 1:2.6. A plethora of peaks from vibrational transitions is furthermore observed across this wavenumber region. In order to unambiguously assign these, we turn to the electrostatic deflection technique.

**Figure 3:**
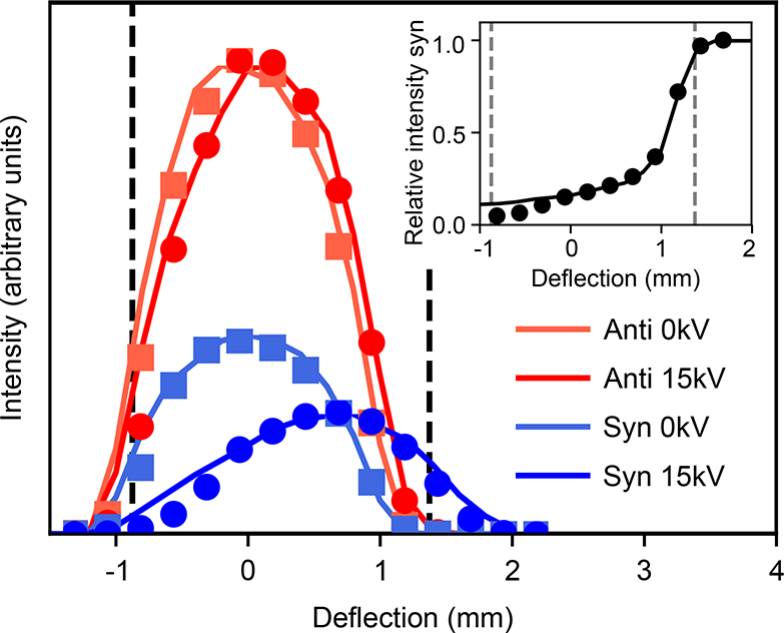
Measured molecular beam intensity of *syn* (blue) and *anti* 3-aminophenol (red) as a function of laser height (data points), and matching trajectory simulations (solid lines), yielding a rotational temperature of 1.3 K. The inset shows the relative purity of the *syn* conformer across the deflected molecular beam. The vertical lines show the positions at which the resonance enhanced multiphoton ionization spectra for the pure *syn* (right) and *anti* (left) conformers were measured.

Applying a potential difference across the deflector electrodes leads to a shift and dispersion of the species within the molecular beam, depending on the underlying effective dipole moment to mass ratio. The deflection of both *syn* and *anti* conformers was measured at potential differences of 0 and 15 kV by measuring the intensity of the respective origin bands while scanning the laser height. In Figure 3, the resulting beam profiles as a function of laser focus position are shown (data points), while the solid lines indicate the profiles extracted from numerical trajectory simulations for a rotational temperature of 1.3 K. A clear spatial separation of the conformers was observed and was in very good agreement with simulations. At a height of ∼1.4 mm above the center of the beam we observed almost exclusively molecules in the *syn* conformation, while ∼0.8 mm below the center the molecular beam was almost fully depleted for the *syn* conformer. The inset of Figure 3 shows the fractional intensity of the *syn* conformer across the molecular beam deflection coordinate for a potential difference of 15 kV across the deflector. The dashed lines indicate the positions where the most pure samples of the *syn* and *anti* conformers can be probed, while still having reasonable intensity.

**Figure 4:**
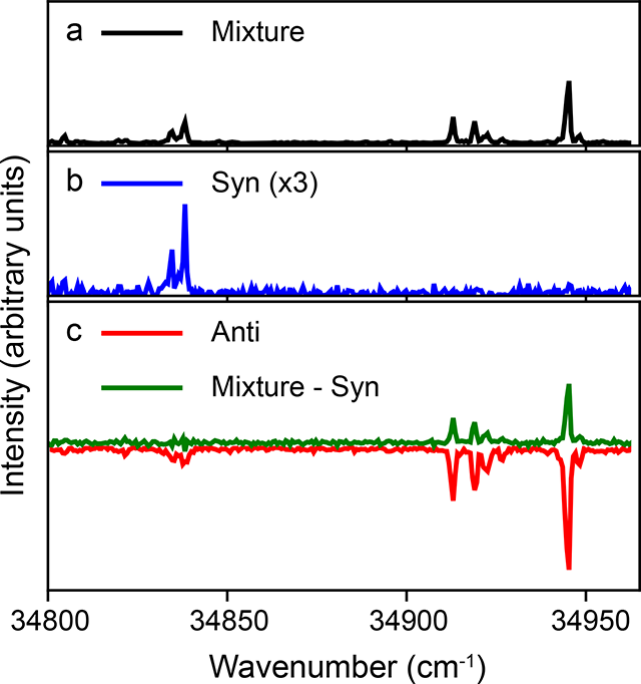
Zoom-in of the spectral region 34 800 cm^–1^ to 35 000 cm^–1^ for the spectra shown in Figure 2, highlighting the purity of the produced molecular beams and the trivial assignment of lines to conformers.

REMPI spectra for these pure *syn* and *anti* conformers are shown in Figure 2b,c. We furthermore show in panel c the spectrum collected in the undeflected molecular beam (panel a) with the pure *syn* spectrum (panel b) subtracted. It is clear from these spectra that the deflection technique creates conformer-pure samples, making assignment of lines to individual conformers trivial. This is further highlighted in Figure 4, which shows a detailed view of the spectral region 34 800 to 35 000 cm^–1^. The collected spectrum for the *anti* conformer contains a small contribution from the *syn* conformer, but the much reduced intensity still allows unambiguous assignment. Moreover, the very pure spectrum obtained for the *syn* conformer allows reconstruction of the pure-*anti* spectrum by subtracting the *syn* contribution from the collected mixture spectrum, as shown by the green line in Figure 2 and Figure 4. This further confirms that only two isomeric forms are present in the molecular beam. These fully conformer-resolved spectra based on spatial separation make it very easy to unambiguously assign all peaks to one of the conformers.

A full list of the observed spectral lines for the *syn* and *anti* conformers, along with tentative assignments, are given in the Supporting Information. Assignments are based on previously published IR–UV double resonance spectroscopy experiments performed at the FELIX free-electron laser facility and the corresponding high-level theory,^20^ as well as a previously published UV REMPI spectrum.^19,21^ We observe several features that have not been experimentally observed or assigned previously. For example, we observe clear spectral features due to an aromatic ring out-of-plane bend at 217 and 227 cm^–1^ for the *syn* and *anti* conformers, respectively. This was not previously observed but agrees well with published theory.^20^ We furthermore observed strong bands at 322 cm^–1^ (*syn*) and 314 cm^–1^(*anti*), which theory suggests are due to OH wagging or NH_2_ torsion. The previous experiments at FELIX suggest that the OH wagging shows a significantly stronger response, and we tentatively assign the observed bands to this. Interestingly, in the S_1_ state probed here, the band belonging to the *syn* conformer appears higher in energy, whereas in the S_0_ state accessed by double-resonance experiments, the *anti* band is higher (307 cm^–1^ for *syn* and 316 cm^–1^ for *anti*(20)). For the *syn* conformer, we further observe a strong band at 639 cm^–1^, which we tentatively assign to the overtone of this transition. Moreover, the 0 → 2 and 0 → 3 overtones of the NH_2_ wag are observed for the *syn* conformer at 424 and 732 cm^–1^, agreeing well with previous experiments and calculations.^20^ In the spectrally congested region of 400–500 cm^–1^, we confirm several bands in both conformers that have been assigned to a strong mixing of the **6a**/**6b** in-plane bending modes, caused by a Fermi resonance or the Duschinsky effect.^21^ It is clear that the trivial and unambiguous assignment of lines to a particular isomer adds a valuable new tool for isomer-specific spectroscopy, in particular for the often closely spaced torsional modes that are difficult to assign based on theoretical considerations alone.

The presented technique of isomer-resolved spectroscopy by electrostatic separation has several advantages over the established approach based on double resonance schemes. It requires only a single table-top laser source, whereas double resonance schemes always require (at least) two tunable laser sources. Moreover, schemes based on hole burning frequently require IR “burn” pulses with significant pulse energy to achieve sufficient depletion, which are not widely available and restricted to a few IR free-electron laser sources around the world. The electrostatic separation approach furthermore allows assignments of spectral lines to individual conformers without relying on calculations, i.e., without any *a priori* knowledge. Which spectral features originate from the same conformational structure can be determined purely from the experimental data. Assigning features to a particular structure requires only the calculation of the associated dipole moments and Stark effect.

Our methodology is widely applicable and requires only that molecules can be entrained into a rotationally cold molecular beam, and that the isomers have a sufficient difference in dipole moment. For typical aromatic molecules with masses up to a few hundred Daltons, a difference in dipole moment of ∼1 D and a rotational temperature of a few Kelvin is sufficient to obtain a pure sample of the more polar isomer. The latter is routinely achieved in pulsed supersonic molecular beams,^22^ and also within reach of new approaches to buffer-gas cooling that could provide significantly slower molecular beams, and hence increased electrostatic deflection.^23^ We also note that, in principle, the production of isomer-pure samples is not required, as long as samples with different (but known) population ratios can be produced, the individual spectra can be recovered through a global analysis.^24^

While we demonstrated here the collection of pure spectra for individual conformers (rotational isomers), the approach is generally applicable to all species within a cold molecular beam, from other forms of structural isomerism to solvent–solute clusters formed during expansion.^14,25^ For example, it would allow the separation and individual study of different tautomeric structures such as those of cytosine, which is known to exist in six tautomeric forms, of which three typically are present in a molecular beam.^26^ These three tautomers have dipole moments of 6.15, 4.52, and 3.10 D, which is sufficient to separate and study these structures individually. In general, spatial separation of isomers in a molecular beam adds isomer specificity to techniques that by themselves lack the spectral resolution needed to distinguish isomers. This has been demonstrated for molecular collision experiments^1^ and is furthermore of use to any ultrafast dynamics experiment, which, due to the inherent bandwidth in a femtosecond pulse, cannot distinguish isomers spectroscopically.

## IV Conclusion

We presented here a technique for performing isomer-resolved spectroscopy using only a single table-top laser, and which allows the assignment of transitions to an isomer without relying on theoretical predictions or double resonance schemes. This was demonstrated for the *syn* and *anti* conformers of 3-aminophenol, and fully isomer-resolved REMPI spectra were presented and assigned, including several previously unobserved lines. The approach is widely applicable to any polar molecule with a sufficient difference in dipole moment between the isomers. It is furthermore not limited to single-color REMPI spectroscopy as adopted here, instead the physical separation of the isomers in principle allows probing by any laser-based spectroscopic method. When using REMPI, it requires only a single resonance process, in contrast to hole-burning approaches that are double-resonance spectroscopies. Its implementation requires only a static electric field and allows one to add isomer specificity to any molecular-beam-based spectroscopy experiment.

## Supporting Information Available

Detailed description of the experimental setup and list of observed spectral lines (PDF)
