# Supplementary material for: Single-Color Isomer-Resolved Spectroscopy
Source: J Phys Chem A. 2022 Jun 1;126(23):3811–5. doi: 10.1021/acs.jpca.2c02277 (PMC9207891; doi:10.1021/acs.jpca.2c02277)
Supplement: Supplementary file 1 — jp2c02277_si_001.pdf [file jp2c02277_si_001.pdf]

Supporting Information:
Single-Color Isomer-Resolved Spectroscopy

Grite L. Abma,[1] Dries Kleuskens,[1] Siwen Wang,[1] Michiel Balster,[1] Andre van Roij,[1] Niek Janssen,[1] and Daniel A. Horke[1,*]

[1]*Radboud University, Institute for Molecules and Materials, Heijendaalseweg 135, 6525 AJ Nijmegen, The Netherlands*

(Dated: May 16, 2022)

* Corresponding author. Email: d.horke@science.ru.nl

I. Technical description of molecular beam apparatus

Experiments were conducted on a custom build molecular beam spectrometer featuring a rod-and-trough type ('a-type') electrostatic deflector[1] and velocity-map imaging (VMI) detector,[2] operated here in ion time-of-flight mode.

Solid 3-aminophenol was placed inside the heated sample compartment of an Even-Lavie molecular beam valve, heated to 140 ° C. The resulting molecular beam was first skimmed 125 mm downstream from the nozzle with a 2 mm diameter skimmer (all skimmers from Beam Dynamics Inc.), and again 256 mm from the nozzle with a 1 mm diameter skimmer. The produced molecular packet enters the 30 cm long electrostatic deflector 355 mm downstream of the nozzle. A cross-section of the deflector, indicating the typical electric fields inside, is shown in Figure S1. After the deflector the molecular beam is skimmed once more 681 mm downstream of the nozzle with a 1.5 mm diameter skimmer.

The molecular beam is probed at the center of the VMI spectrometer, which extracts ions perpendicular to the molecular beam and laser propagation direction. The VMI spectrometer consists of 3 stainless steel plates (130 mm outer diameter, 2 mm thickness) placed 25 mm apart. The second and third electrodes have central apertures of 30 mm diameter. Ions are accelerated onto a dual micro-channel plate (MCP) detector (Photonis Inc., 40 mm diameter, 5 µm pore size), that is also equipped with a P43 phosphor screen (not used in the present experiments). Ion signal is read out from the back MCP and feed into an analog-digital converter (Spectrum Instrumentation) for analysis.

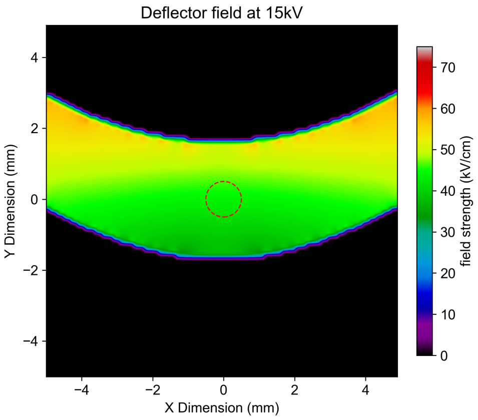

FIG. S1. Cross-section of the electrostatic deflector, and indication of the electric field inside for an applied potential difference of 15 kV. The dashed circle indicated where the molecular beam enters the deflector.

II. List of observed spectral lines and tentative assignments

Table S1 and Table S2 list the observed spectral lines for the *syn* and *anti*-conformers, respectively. Tentative assignments based on published experimental spectra and high-level theory are also indicated.

Absolute Frequency (cm^{-1})	Shift (cm^{-1})	Rel. Int.	Tent. Assignment
34106.8	0	1	
34323.7	216.9	0.34	C-C-C o.p. bend **10a** or **10b**
34429.0	322.2	0.4	OH wag or NH_2 torsion
34462.7	355.9	0.28	-
34508.0	401.2	0.47	-
34531.2	424.4	0.64	NH_2 wag overtone 0-2
34540.9	434.1	0.29	overtone **10a** or **10b**
34550.9	444.1	0.2	vibr. mixing **6a**/**6b**
34559.5	452.7	0.36	vibr. mixing **6a**/**6b**
34566.1	459.3	0.25	vibr. mixing **6a**/**6b**
34579.2	472.4	0.42	vibr. mixing **6a**/**6b**
34586.7	489.9	0.62	vibr. mixing **6a**/**6b**
34597.8	491.0	0.49	vibr. mixing **6a**/**6b**
34680.9	574.1	0.26	Comb. 37(1)+36(1)
34746.0	639.2	0.41	overtone OH wag or NH_2 torsion
34834.8	728.0	0.49	ring breathing $\mathbf{1}^1$
34838.3	731.7	0.32	NH_2 wag overtone 0-3

TABLE S1. Transitions in the spectrum of *syn* 3-aminophenol. All energies are relative to the 0–0 origin at 34107 cm^{-1}. Assignments are based on published double-resonance spectra and high-level theory,[3] and previous REMPI spectra.[4,5]

Absolute Frequency (cm^{-1})	Shift (cm^{-1})	Rel. Int.	Tent. Assignment
34473.3	0	1	
34700.6	227.3	0.3	C-C-C o.p. bend **10a** or **10b**
34787.5	314.2	0.28	OH wag or NH_2 torsion
34913.0	439.7	0.4	vibr. mixing **6a/6b**
34918.8	445.5	0.32	vibr. mixing **6a/6b**
34922.6	449.3	0.17	vibr. mixing **6a/6b**
34945.1	471.8	0.92	vibr. mixing **6a/6b**

TABLE S2. Transitions in the spectrum of *anti* 3-aminophenol. All energies are relative to the 0–0 origin at 34473 cm^{-1}. Assignments are based on published double-resonance spectra and high-level theory,[3] and previous REMPI spectra[4,5]

[1] de Nijs, A. J.; Bethlem, H. L. On deflection fields, weak-focusing and strong-focusing storage rings for polar molecules. *Phys. Chem. Chem. Phys.* **2011**, *13*, 19052–8.

[2] Eppink, A. T. J. B.; Parker, D. H. Velocity map imaging of ions and electrons using electrostatic lenses: Application in photoelectron and photofragment ion imaging of molecular oxygen. *Rev. Sci. Instrum.* **1997**, *68*, 3477–3484.

[3] Yatsyna, V.; Bakker, D. J.; Feifel, R.; Rijs, A. M.; Zhaunerchyk, V. aminophenol isomers unraveled by conformer-specific far-IR action spectroscopy. *Phys. Chem. Chem. Phys.* **2016**, *18*, 6275–6283.

[4] Shinozaki, M.; Sakai, M.; Yamaguchi, S.; Fujioka, T.; Fujii, M. $S_1 \leftarrow S_0$ electronic spectrum of jet-cooled m-aminophenol. *Phys. Chem. Chem. Phys.* **2003**, *5*, 5044–5050.

[5] Xie, Y.; Su, H.; Tzeng, W. B. Rotamers of m-aminophenol cation studied by mass analyzed threshold ionization spectroscopy and theoretical calculations. *Chem. Phys. Lett.* **2004**, *394*, 182–187.
